# Notes concerning the peritonitis of urinary aetiology

**Published:** 2008-02-25

**Authors:** D Mischianu, 0 Bratu, C Ilie, V Madan

**Affiliations:** Urology Clinic – Central Military Emergency Universitary Hospital ‘Dr. Carol Davila’Romania

## Abstract

Urinary peritonitis (the uroperitoneum) is categorized as a difficult to diagnose clinical entity due to its poor manifestations.

Vesical trauma following pelvis bone fracture is the most frequently involved in the uroperitoneum aetiology, followed by spontaneous vesical rupture and intraoperative iatrogenic lesions.

One of the most important and constant signs that can occur is diffuse abdominal tension, without tenderness.

The imagistic procedure that sets the diagnosis is retrograde cystography showing intraperitoneal urine effusion.

Vesical rupture is a surgical emergency.

The uroperitoneum is a particular type of peritonitis that has hidden and misleading symptoms which can delay the diagnosis long enough to endanger the patient's life.

The truth about urinary peritonitis (or uroperitoneum as surgeons call this clinical entity) is that it is – restating the motto – a ‘peculiar’ peritonitis! We will try to explain in the following paragraphs what this peculiarity consists of. Simply defined as the presence of urine in the peritoneal space, the uroperitoneum conceals many characteristics although at first sight can be enclosed in the rich category of causes for secondary, let us say ordinary peritonitis (urinary aetiology peritonitis: rupture of high urinary pathways or of urinary bladder). It represents through its content and clinical manifestations a rarely encountered and problematic type of pathology. There are not few the times when we see in medical literature strange cases or let us call them less than usual cases, not only by being spectacular but by being difficult to diagnose. There are not few the times when we confront with situations in which the patient's state makes us think although laboratory findings are within ‘loose normal limits’!

Without exaggerating, we believe that there are cases when many experienced surgeons can not tell for certain if they are facing an uroperitoneum. Without any doubt, the diagnosis of the uroperitoneum is a difficult one and requires clinical experience (if there is seen such a thing) or flair.

We have to particularly mention that not long ago (the very reason behind this article) we encountered such a case. It wasn't the first time we had seen in our practice a patient with urinary peritonitis. A woman of 52 years of age, a farmer in the Focsani county (observation sheet no. 49104 from 21.01.2008), presented to our hospital at one of the medical clinics then admitted directly in the ICU due to her altered general status and acute renal failure: oligo-anuria – 300ml in 24 hrs, serum potassium of 7.7 mEq/l, creatinine of 8.5mg/dl etc., without any other symptoms or signs. 24 hours later we were called for a ‘collegial’ consult in order for us to insert an autostatic double JJ stent (the well known Cook catheter) or a nephrostoma, something?!. . . (She was anuric after all).

We examined the patient taking into account more than biochemical lab values and CT images (dubitative interpretation). We noticed that she had diffuse abdominal tension and vague hypogastrium pain. Retrograde cystography (Figure 1) showed the flow of contrast substance in the peritoneal space. To be noted that we made this investigation not due to the suspicion of uroperitoneum but because of our previous experience, years ago, of a somewhat similar case: a 35 year old autistic man, sheared between two carriages of a circulating tram, without pelvis fracture but with urinary  bladder rupture and consequent uroperitoneum.

**Figure 1:**
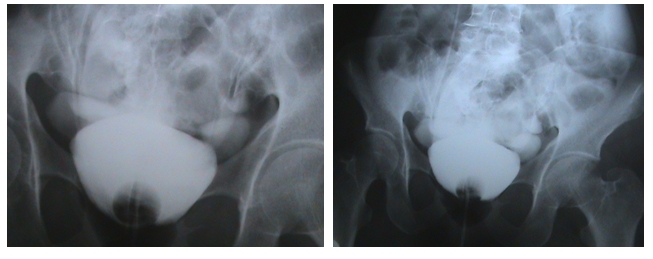
Retrograde cystography – the flow of contrast substance in the peritoneal space.

We intervened surgically through an anterior abdominal incision and intraoperatively, an old an important vesical lesion was seen, on a bladder that looked ‘withered’, well fixed on an intestinal loop, which explained the almost missing symptoms. Without bringing new information in addition to the ones already reported in literature, we will note in the following, a few aspects on urinary peritonitis, with an intent of more than attracting attention towards this type of pathology

Urine may get between intestinal loops by effraction through one of the urinary tract organs and the most frequently involved is the urinary bladder, through the rupture of its peritoneal side. Theoretically, the uroperitoneum can appear also through renal or urethral trauma but, considering their retroperitoneal location, causing agents must have a more complex and aggressive mechanism. (Figure 2)

**Figure 2:**
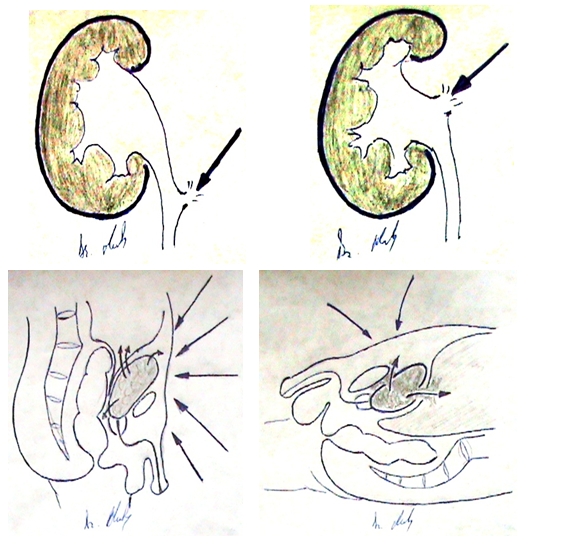
Manner of action of the traumatic agent with subsequent urethral and renal lesions followed by flow of urine and Hypogastrium trauma with intra– and extra peritoneal urine effusion

As a consequence, urinary bladder ruptures can be spontaneous, traumatic or iatrogenic.Spontaneous ruptures presume in principle the existence of a thin vesical wall, an immunosuppressant status or a lack in body proteins, such as consumption diseases. Quite a few at a first glance, these pathological situations which lead to the spontaneous effraction of the bladder make us think that they are least probable to be juxtaposed in order to make the optimal frame that causes urinary peritonitis. Nevertheless, it happens!

Most frequent vesical ruptures appear in situations like chronic alcohol intakes (drunk man's bladder) [3 ], immunosuppression, cirrhosis, diabetes, tuberculosis, scarred or tumoral bladders, when there are large vesical diverticula or chronic vesical obstacles with thin vesical wall. The question of a determining factor of vesical rupture was raised, in spite of the mentioned associated pathology. Most of the times there is a traumatic mechanism involved, brought to light by a very detailed history but there are cases in which no trauma can be traced.

Vesical ruptures are induced mainly by action of the injury agent directly on the inferior abdomen. This determines a great and sudden pressure on the abdominal wall which spreads to the bladder, projects itself on the posterior wall of the pelvis and due to its resistance and comes back towards the bladder (anterior), giving it a counter blow [2].

To this mechanism, a series of favourable conditions which may contribute to the extent of the lesions can be variably added.

The amount of urine present inside the bladder at the moment of injury plays an important part. If the bladder is empty, it can be torn only through direct impact of the trauma agent on its walls because of its deep location in the pelvis and its protection by pelvic bones. Full bladder comes out of that protection and becomes an intraperitoneal organ. Vesical wall grows thinner in proportion with the quantity of urine and the flexibility of its muscular fibres decreases. Anatomical areas with least resistance are the superior and posterior walls.

Vesical pathologies which determine a decline in wall strength (inflammatory, tumoral, scaring processes) favour surprisingly serious lesions following minor traumas.

Neurological lesions of vesical wall lower its ability of distension and also muscle tone and consequently allow an accumulation of a great amount of urine. It is the case of neurogenic bladder patients. 

Last but not least, vesical ruptures can be accidentally produced during surgery of the pelvic area or during endoscopy procedures when, due to vesical wall lesion, the washing endovesical fluid effuses in the peritoneal cavity and produces an iatrogenic uroperitoneum (the patient's abdomen grows ‘second by second’).

## Physiopathology

The intimate substratum of urinary peritonitis symptoms is common to every peritonitis but noting that it depends greatly with the quantity and quality of urine, if we're allowed to say it like this. When a sterile urine effuses, the symptoms are minimal, hidden and become clinically obvious when this urine becomes infected. In the same way, the clinical suffering and the expressed symptoms can be seen when urine is infected per primam.

Many local defence mechanisms come to aid against infection and other immune as well. Mechanical ones belong to the structural anatomy and to different characteristics of the peritoneum. Inside the immune mechanisms are cellular components, serum ones, unspecific immunity and the complement system.

Confinement of infection is realised through direct absorption of bacteria in the lymphatic system, their fagocytosis and formation of intraperitoneal abscesses [4, 5]. The systemic response to bacterial aggression lies in the release of physiological messengers of inflammation: cytokines (TNF alpha, IL–1, IL–6, interferon, etc) and cellular effectors (neutrophils, monocytes, macrophages, endothelial cells). These activated cellular effectors lead to the synthesis and secretion of new cytokines and secondary inflammation mediators (prostaglandins, leucotriens, tromboxans, trombocyte activating factor etc) [4, 5].

Loss of local control is clinically identified with SIRS –systemic inflammatory response syndrome – which subsequently evolves toward MODS –multiple organ dysfunction syndrome, which will not be detailed here. Most times, newly diagnosed uroperitoneum cases are comprised in the above mentioned situations [4, 5].

Finally, in serious and delayed treatment cases, multiple organ dysfunction becomes a sombre entity called CHAOS (Cardiovascular shock, Homeostasis, Apoptosis, Organ dysfunction, immune Suppression), with minimal chances of survival [4, 5].

## Clinical Manifestations

Symptoms of vesical trauma and namely of urinary peritonitis are not specific.

In the case of a pathological bladder, a surprising discordance may appear between the low force of the injury agent and the emergence of vesical lesions (a minor trauma may determine lesions of maximum gravity). Some other times, the tear of vesical wall is distanced from the moment of the accident, representing the so-called ‘two–stage vesical rupture’ (a temporary obstruction of the lesion with omentum or intestinal loops which determines tardy solution of continuity) [1].

The presence of sterile urine in the peritoneal space can be tolerated for many days, with faded, inconclusive local symptoms. In this situation, alteration in general wellbeing and imbalance of electrolytes are more obvious etc. Perforations and ruptures of urinary bladder usually begin with a violent pain, located in the pelvis or the perineum and are relatively constantly accompanied by vesical and rectal tenesmes. If urine emission continues to be spontaneous, it is accompanied by polakiuria, dysuria, haematuria, painful urine emission which is also weak, discontinuous and with a sensation of incomplete evacuation [1, 7]. In principle, the manifestations of uroperitoneum can be organised into the following symptoms and signs, one or two being present in common practice, thus making diagnosis difficult  [1, 2]:

- actual signs of hypogastric lesions (excoriations, haematomas);
- hypogastrium pain due to trauma;
- increase of abdomen volume, a consequence of urine accumulation in the peritoneal cavity (ascites); intraperitoneal fluid can be perceived by percussion, inspection and palpation of the abdomen;
- muscle contraction situated at first in the pelvis takes over the entire superior abdomen;
- abdominal pain irradiating to the shoulder, consecutive to urine accumulation under the diaphragm, with subsequent irritation of the phrenic nerve (Kehr sign);
- peritonitis signs in the first hours after vesical tear if urine is highly infected (purulent) or after a few days if urine is initially sterile;
- abdominal distension caused by paralytic ileus which installs slowly and aggravates itself continuously;
- partial or complete ceasement or urine emission, with a slow, passive flow of urine in the peritoneum;
- peritoneal irritation signs on rectal exam; The diagnosis is, as we mentioned before, pretty difficult because the uroperitoneum is a hidden disease, with tardy manifestations most of the times. Diagnosis is based on traumatic history (pelvic fracture for example associates vesical tears in 90% of cases), previous endoscopy manoeuvres and their juxtaposed pathology and presence of more than one of the above mentioned signs.

In a book, the only one about this subject published in Romania and also a beginning of trade cornerstone for experienced surgeons – ‘Surgical exploration of abdomen’ by Dr. Dan Gerota (Medical Publishing House 1969, first edition, page 70, same publishing house 1987, second edition) [6] – the unsurpassed surgeon and teacher pointed out: ‘Other effusions (peritoneal effusion, authors note) are rarely encountered and more difficult to diagnose, especially urine, when mixed with blood or in low quantity (the case of urether lesions). Even in the case of a vesical lesion, if empty during trauma, in the case of little continuity fluid, with outlet or situated in an extraperitoneal area, we encounter the same difficulties.’

## Imagistic explorations

**Figure 3:**
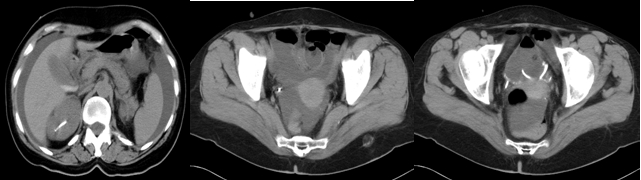
Computer tomography (without contrast) – massive intraperitoneal effusion (uroperitoneum by spontaneous vesical tear); near the urinary bladder an intestinal loops adheres to the lesion

- reno–vesical radiograph does not bring many information but can show pelvic bones fractures and eventually detect an area of increased density in the hypogastrium or pelvis consecutive to the accumulation of urine or blood;
- intravenous urography shows morphological and functional integrity of the superior urinary tract and vesical rupture will appear in late stages or on the cystograms by contrast substance effusion in the peritoneal cavity;
- retrograde cystography is possibly the most important diagnostic method of vesical rupture and consists in the instillation of a certain quantity of contrast substance in the urinary bladder by means of an urethral–vesical catheter; many films can be obtained, in many views that will show the effusion of contrast substance among intestines;
- abdominal ultrasonography shows fluid accumulation in the peritoneal cavity, similar to ascites fluid;
- abdominal and pelvic CT and MRI show the presence of fluid in the peritoneal cavity and if they are done with contrast substance, its effusion can be observed when it reaches the urinary bladder (Figure 3)

Information offered by ultrasound and imaging explorations are doubtful and most often the interpretation offered by our specialist colleague is charged with a high, subjective and lacking involvement dose (caused for example by ‘the presence of air in between loops, numerous artefacts’ and so on). In the case that we presented their help was minimal at most.

Urethral cystoscopy is not recommended because the risk of aggravating the lesions is high (washing fluid will effuse in the peritoneum and become an additional factor of infection). If the manoeuvre is done nonetheless, the actual lesion, where intestinal loops or parts of the epiploon enter, can be seen inside the bladder [7, 8].

## Treatment

The treatment of vesical ruptures and uroperitoneum is primarily surgical, of outmost urgency and is made of a median abdominal incision (preferably xifo–pubian for a wider approach) through which the entire abdominal cavity and pelvis are explored. The vesical lesion is sutured, eventually in double layer with the excision of anfractuous vesical margins and the bladder is drained efficiently in order to ensure integrity of the future suture. An inspection of the entire cavity is done, false membranes are cleaned if present, the peritoneum is washed with a dextrane or saline solution and drained with one or more drainage canules, correctly placed [7, 8].

When the agent of injury has a big enough force and determines an almost complete destruction of the bladder, enlargement enteral cytoplasties can be done.

Supportive postoperative treatment plays a very important part. Antibiotics must have a large spectrum, eventually be against anaerobes and in close to maximum dose. Gastric protection (antiacids to prevent stress ulcers) is imposed along with electrolyte and acid–base balance, anticoagulants to avoid disseminated intravascular coagulation, amino acids, human albumin or proteins to strengthen the immune system.

This article does not follow known and asked for by the editor's author recommendations which is to say it will not conclude with anything.

We wrote it because we wished to remind that the uroperitoneum is a type of peritonitis that has hidden manifestations which can greatly delay diagnosis so that patient's life is jeopardised.Clinical signs at presentation are most often few and common, one of which is muscle contraction without tenderness in the absence of subumbilical trauma.

As it is often said, to make a good diagnosis, one must think of it! From here on to the motto: ‘Qui bene diagnoscit, bene curat!’ only one step must be taken…
